# Supplementary material for: Beyond Carcinoembryonic Antigens: The Role of CA-125 and CA-199 in Predicting Prognosis of Lung Adenocarcinoma
Source: Cancers (Basel). 2025 Apr 30;17(9):1517. doi: 10.3390/cancers17091517 (PMC12070945; doi:10.3390/cancers17091517)
Supplement: Supplementary file 1 [file cancers-17-01517-s001.zip › cancers-3556790-supplementary.pdf]

Table S1. Tumor markers in patients with adenocarcinoma at different stages

	**Stage I and II (N = 550)**	**Stage III (N = 56)**	**Stage IV treated with EGFR TKI (N = 527)**	***P* for trend**
CEA (log)	0.54 ± 0.73 (N = 506)	2.05 ± 1.73 (N = 55)	3.03 ± 2.18 (N = 319)	<0.001
CA125 (Log)	2.81 ± 0.59 (N = 92)	3.91 ± 1.27 (N = 28)	4.35 ± 1.47 (N = 213)	<0.001
CA153 (Log)	2.28 ± 0.46 (N = 45)	2.35 ± 0.48 (N = 2)	3.38 ± 1.39 (N = 51)	<0.001
CA199 (Log)	2.52 ± 0.81 (N = 142)	2.86 ± 1.23 (N = 32)	3.35 ± 1.72 (N = 252)	<0.001

Notes: Data are presented as mean ± standard deviation.

Abbreviations: EGFR, epidermal growth factor receptor; TKI, tyrosine kinase inhibitor; CEA, Carcinoembryonic antigen; SCC, Squamous cell carcinoma antigen; CA, Cancer antigen
